# Identification and Comparative Analysis of ncRNAs in Human, Mouse and Zebrafish Indicate a Conserved Role in Regulation of Genes Expressed in Brain

**DOI:** 10.1371/journal.pone.0052275

**Published:** 2012-12-20

**Authors:** Zhipeng Qu, David L. Adelson

**Affiliations:** School of Molecular and Biomedical Science, The University of Adelaide, Adelaide, Australia; National Institutes of Health, United States of America

## Abstract

ncRNAs (non-coding RNAs), in particular long ncRNAs, represent a significant proportion of the vertebrate transcriptome and probably regulate many biological processes. We used publically available ESTs (Expressed Sequence Tags) from human, mouse and zebrafish and a previously published analysis pipeline to annotate and analyze the vertebrate non-protein-coding transcriptome. Comparative analysis confirmed some previously described features of intergenic ncRNAs, such as a positionally biased distribution with respect to regulatory or development related protein-coding genes, and weak but clear sequence conservation across species. Significantly, comparative analysis of developmental and regulatory genes proximate to long ncRNAs indicated that the only conserved relationship of these genes to neighbor long ncRNAs was with respect to genes expressed in human brain, suggesting a conserved, ncRNA cis-regulatory network in vertebrate nervous system development. Most of the relationships between long ncRNAs and proximate coding genes were not conserved, providing evidence for the rapid evolution of species-specific gene associated long ncRNAs. We have reconstructed and annotated over 130,000 long ncRNAs in these three species, providing a significantly expanded number of candidates for functional testing by the research community.

## Introduction

Protein-coding genes account for only a small proportion of vertebrate genome complexity, specifically, only ∼2% of the human genome [1]. With better and more sensitive methods for studying gene expression, such as genome tiling arrays and deep RNA sequencing, we now know that vertebrate “RNA-only” transcriptomes are much more complex than their protein-coding transcriptomes [2], [3], [4], [5]. Studies of some vertebrate genomes have indicated that there are tens of thousands of ncRNAs (non-coding RNAs) [6], [7], [8], including structural RNAs, such as ribosomal RNAs, transfer RNAs and small non-coding regulatory transcripts such as siRNAs (small interfering RNAs), miRNAs (micro RNAs) and piRNAs (piwi-interacting RNAs) [9]. In addition to these well-characterized ncRNAs, there are a substantial number long ncRNAs, only a few of which have been functionally characterized [10], [11], [12], [13], [14].

The few functionally characterized long ncRNAs have various regulatory roles ranging from gene imprinting [15], [16], to transcriptional activation/repression of protein-coding genes [17], [18]. Specific long ncRNAs have been found with roles in neural development [19] and cell pluripotency [20], [21]. Long ncRNAs have also been implicated in pathological processes resulting from aberrant gene regulation [13], [22], [23]. But not all long ncRNAs are the same and a number of different methods have been used to discover and annotate them. Guttman *et al.* identified thousands of lincRNAs (large intervening/intergenic non-coding RNAs) in mouse using chromatin signatures [10], and Khalil *et al*. extended the catalog of human chromatin-signature-derived lincRNAs to ∼3,300 using the chromatin-state maps of 6 human cell types [11]. Many more lincRNAs have been reconstructed from RNA-seq data from multiple sources in human, mouse and zebrafish [12], [14], [24] and over a thousand long ncRNAs, some of which showed enhancer-like activity, were characterized based on GENCODE annotation [25].

Extrapolation from the limited set of experimentally validated long ncRNAs supports the idea that long ncRNAs are a “hidden” layer of gene regulation. Two lines of evidence supporting this view are their (modest) level of evolutionary sequence conservation and spatial association with regulatory genes. In this report we present the first systematic and methodologically comparable evolutionary analysis of ncRNAs.

In order to determine the full extent of evolutionary conservation of ncRNAs, we used a pipeline built for identifying bovine ncRNAs, particularly long ncRNAs, at genome scale from public EST (Expression Sequence Tag) data. By using ESTs, we were able to get comprehensive datasets of long ncRNAs from both sexes, in many different tissues, cell types, developmental stages, and experimental treatments. In this report we have used this pipeline to analyse all publically available human, mouse and zebrafish ESTs and we present the first global and systematic comparative analysis of non-protein-coding transcriptomes across different species.

We have found large numbers of novel long ncRNAs, many of which originate from the flanking regions of protein-coding genes. Furthermore, we have also shown that gene flanking, intergenic RNAs show sequence conservation compared to non-transcribed genomic regions and are preferentially found near regulatory/developmental protein-coding genes in a species-specific fashion.

## Results

### 1 Genome-wide Exploration of ncRNAs from Human, Mouse, and Zebrafish ESTs

We used a previously described pipeline [26] to screen non-protein-coding transcripts from all publically available human, mouse and zebrafish ESTs and identified over 130,000 ncRNAs (Table 1 and Table S1, http://share:sharingisgood@genomes.ersa.edu.au/ncRNA_pub/). The large numbers of predicted long ncRNAs from human, mouse and zebrafish, together with previously identified bovine ncRNAs, confirm and significantly extend previous reports of pervasive transcription from these four organisms [1], [27], [28].

**Figure 1:**
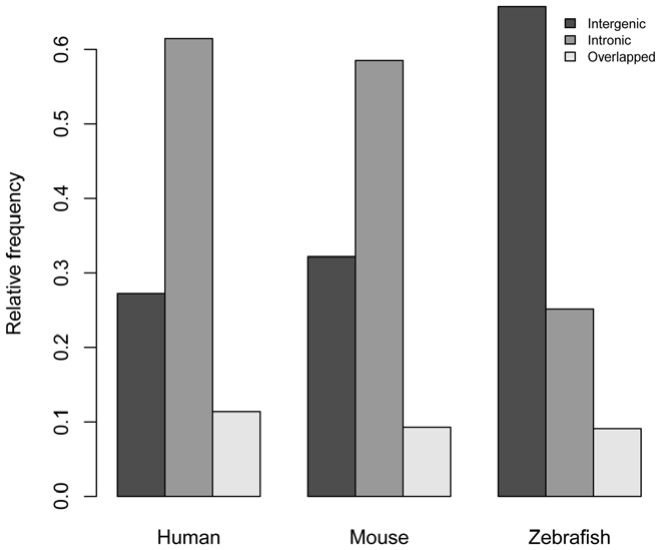
Percentage of intergenic, intronic and overlapped ncRNAs in human, mouse and zebrafish.

Our long ncRNAs fell into 3 categories based on their genomic coordinates with respect to protein-coding genes; intergenic ncRNAs, intronic ncRNAs and overlapped ncRNAs, which overlapped by a small number of base pairs with exons of protein-coding genes [26]. In human and mouse, more than 50% of long ncRNAs were intronic (Figure 1 and Table 2), consistent with previous studies based on other methods [8]. In zebrafish, intergenic ncRNAs were far more numerous than intronic transcripts (Figure 1), but because of the much smaller number of zebrafish intergenic ncRNAs compared to human and mouse (Table 2) it is difficult to be sure that this difference in relative abundance of intergenic ncRNAs is real.

**Table 1:** Summary of procedures for ncRNA identification in human, mouse and zebrafish.

Species	Number of ESTs	Number of assembled transcripts	Mapped to RefSeqs	Mapped to Swiss-Prot	With long ORFs	Putative ncRNAs	Reconstructed ncRNAs
Human*	8,314,483	1,037,755*	44,245*	135,073	130,291	105,994	87,173
Mouse	4,853,460	1,356,763	382,852	3,911	60,342	45,975	36,280
Zebrafish	1,481,936	262,387	117,337	1,828	10,778	11,323	9,877

* Due to the large number of ESTs from human, we ran BLAST for all ESTs against human RefSeqs before assembly and removed all high confident ESTs (coverage >90% and identity >90%). This makes the “Number of assembled transcripts” and “Mapped to RefSeqs” smaller than expected.

**Figure 2:**
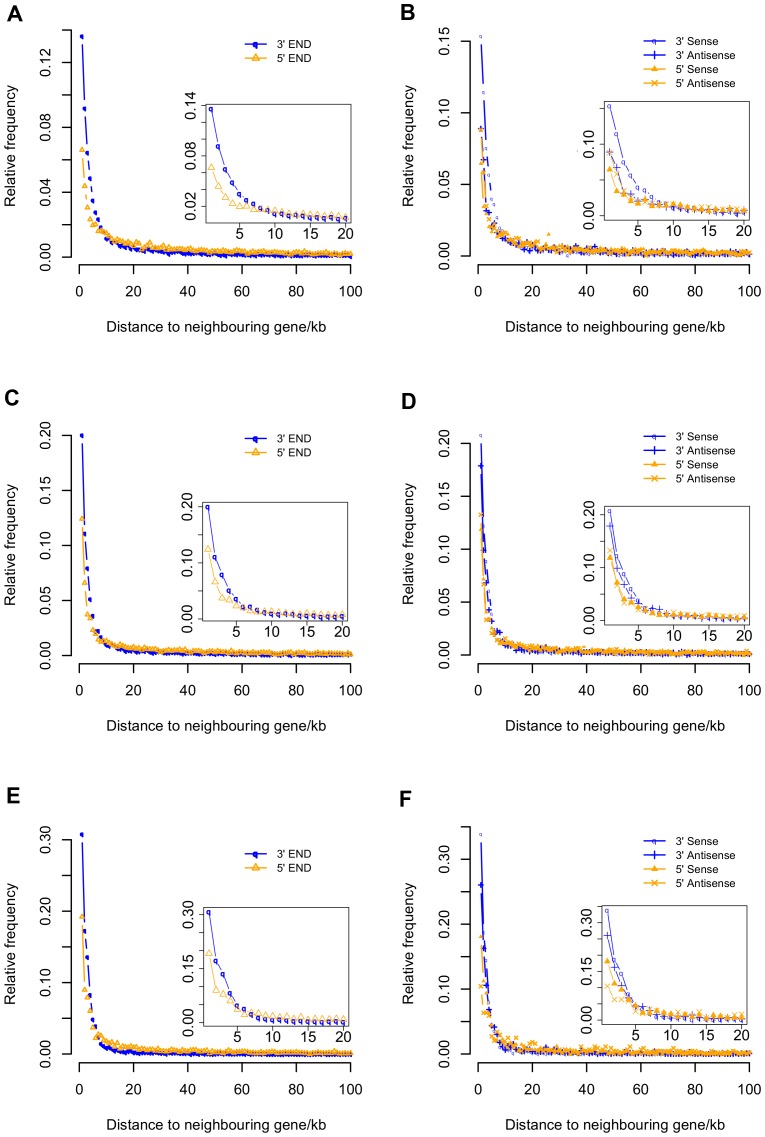
Biased positional distribution of intergenic ncRNAs with respect to neighbor protein-coding genes in human, mouse and zebrafish. The top 2 panels (A & B) are from human, the middle 2 panels (C & D) are from mouse and the bottom 2 panels (E & F) are from zebrafish. A, C and E show the positional distribution of 5′ or 3′ end ncRNAs. B, D and F show the positional distribution of ncRNAs in terms of transcription orientation compared to neighbor genes.

Because many intergenic ncRNAs have been validated as functional elements from different species [10], [12], [14], [25], [29], we focused our analyses on all predicted intergenic ncRNAs. The distribution of intergenic ncRNAs with respect to protein-coding genes was the first question we addressed. In all three species, intergenic ncRNAs showed a biased distribution with respect to protein-coding genes at both 5′ and 3′ ends (Figure 2). This is consistent with our previous observation in cow [26] and previous observations in human and mouse based on tiling array and RNA-seq analyses [30], [31]. Furthermore, we know that many functional transcripts are located in these regions [8], [31].

Larger proportions of sense-strand intergenic ncRNAs were transcribed near the 3′ end of protein-coding genes than antisense ncRNAs in all three species (Figure 2), but the positional distributions of intergenic ncRNAs at the 5′ end of protein-coding genes showed a slightly larger proportion of antisense-strand intergenic ncRNAs, compared to sense intergenic ncRNAs in human and mouse. We considered the possibility that gene-proximate 3′ transcripts were un-annotated UTRs (Untranscribed regions) or alternative transcripts, so we classified these ncRNAs into two subcategories: UTR-related RNAs, that shared high sequence similarity with annotated UTRs or located within 1 kb of protein-coding genes, and “true” intergenic ncRNAs. These results are summarized in Table 2. Some the UTR-related ncRNAs were transcribed from the antisense strand of nearby protein-coding genes, and these may correspond to uaRNAs (UTR-associated RNAs), which are independent transcripts with potential functional significance [32].

**Table 2:** Classification of ncRNAs.

Species	Number of UTR-related ncRNAs	Number of intergenic ncRNAs	Number of intronic ncRNAs	Number of overlapped ncRNAs
Human	3,438	20,268	55,601	10,724
Mouse	2,179	9,490	21,541	4,414
Zebrafish	2,031	4,464	2,514	1,010

### 2 Problems in the Annotation of Long ncRNA Datasets

**Figure 3:**
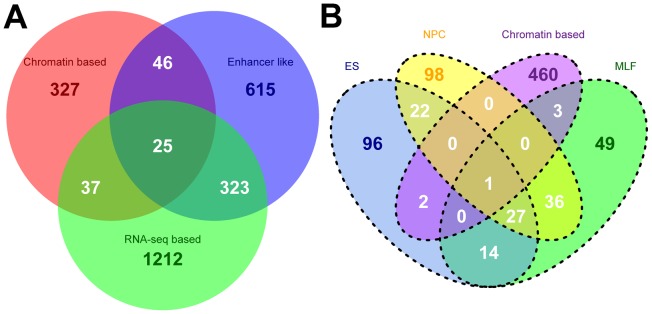
Overlap of our predicted ncRNAs with known human or mouse long ncRNAs from different datasets. A shows the overlap of our ncRNAs with three different human lincRNA datasets. B shows the overlap of our ncRNAs with mouse long ncRNA datasets. “Chromatin based”: lincRNAs identified based on chromatin-state maps [10], [11]. “Enhancer like”: long intergenic ncRNAs identified based on GENCODE [25]. “RNA-seq based”: long ncRNAs identified by reconstruction of RNA-seq data in human. “ES”, “NPC” and “MLF”: long ncRNAs identified by construction of RNA-seq data from 3 different mouse cell types.

**Figure 4:**
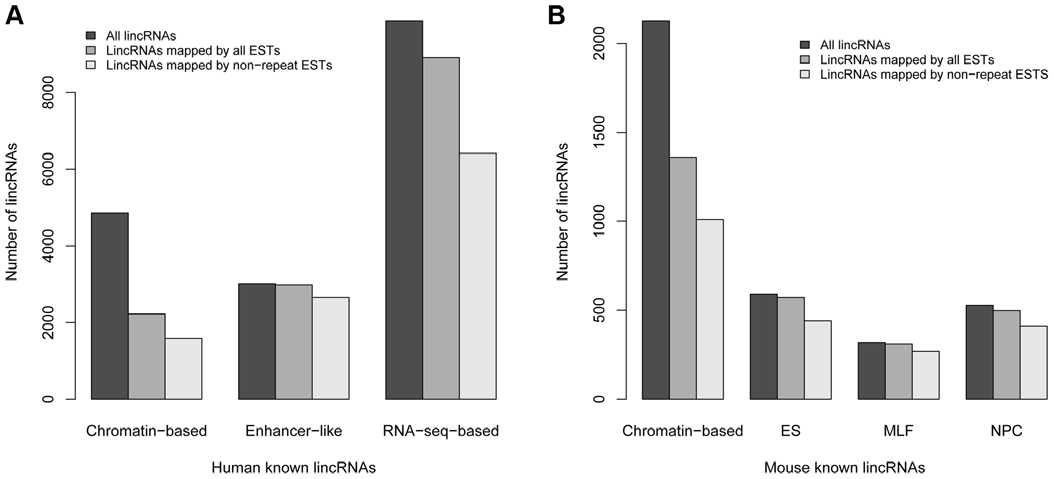
Comparisons of known long ncRNAs mapped by ESTs or non-repeat ESTs in human and mouse. “Chromatin based”: lincRNAs identified based on chromatin-state maps [10], [11]. “Enhancer like”: long intergenic ncRNAs identified based on GENCODE [25]. “RNA-seq based”: long ncRNAs identified by reconstruction of RNA-seq data in human. “ES”, “NPC” and “MLF”: long ncRNAs identified by construction of RNA-seq data from 3 different mouse cell types.

Different methods have been used to identify several classes of long ncRNAs, especially lincRNAs, in human [10], [11], [24], [25], mouse [12] and zebrafish [14]. We compared the genomic coordinates of our long ncRNAs from all available tissues and developmental stages in human, mouse and zebrafish, with previously annotated long ncRNA datasets in order to determine the degree of overlap in ncRNAs identified by different methods. The number of EST-based ncRNAs that overlapped with three different human ncRNA datasets was very limited (Figure 3). Only 2,585 ncRNAs in our dataset had overlap with transcripts in at least one of the three known ncRNA datasets (Figure 3A). 1,597 of them overlapped with ∼16% (2,296 out of 14,353) of RNA-seq-based lincRNAs, and 1,009 overlapped with ∼28% (854 out of 3,011) of enhancer-like long ncRNAs. However, only 435 of them overlapped with ∼10% (508 out of 4,860) of chromatin-based lincRNAs (Table 3). The intersection of all four of these long ncRNA datasets contained only 25 transcripts, but this is to be expected if previously annotated ncRNAs were present in RefSeq, which we used to screen out known genes transcripts from our EST input data. We confirmed the small number of overlaps between our mouse ncRNAs with four other annotated mouse long ncRNA datasets (Figure 3B and Table 3). In order to confirm that this lack of overlap between our results and previously reported long ncRNAs was attributable to this screening process, we aligned them to the ESTs we used as a starting point for ncRNA identification. Depending on the dataset, we found between 46% and 99% of previously reported human ncRNAs in the EST data (Figure 4 and Table S2). We discuss this further below. Because gene models are continuously being revised, we found that some of our non intergenic ncRNAs overlapped with ncRNAs previously described as intergenic (Table 3).

**Table 3:** Overlap of EST-based ncRNAs with previously identified ncRNAs*.

Dataset	Number ofintronic ncRNAs	Number ofoverlapped ncRNAs	Number of UTR-related RNAs	Number of intergenicncRNAs (Percentage**)	In total
Chromatin-based lincRNAs(human)	21	8	15	391/1.93%	435
Enhancer-like long ncRNAs(human)	22	10	32	945/4.66%	1,009
RNA-seq-based lincRNAs(human)	11	19	83	1,484/7.32%	1,597
LincRNAs from ES (mouse)	26	13	15	108/1.14%	162
lincRNAs from MLF (mouse)	40	9	11	70/0.74%	130
LincRNAs from NPC (mouse)	30	14	15	125/1.32%	184
Chromatin-based lincRNAs(mouse)	27	87	59	293/3.09%	466
RNA-seq-based longncRNAs (zebrafish)	16	12	28	105/2.36%	161

* Numbers in this table are shown as our EST-based ncRNAs.

** The percentage is based on the number of all intergenic ncRNAs as shown in table 2.

### 3 Evolutionary Conservation of ncRNAs in Human, Mouse and Zebrafish

**Figure 5:**
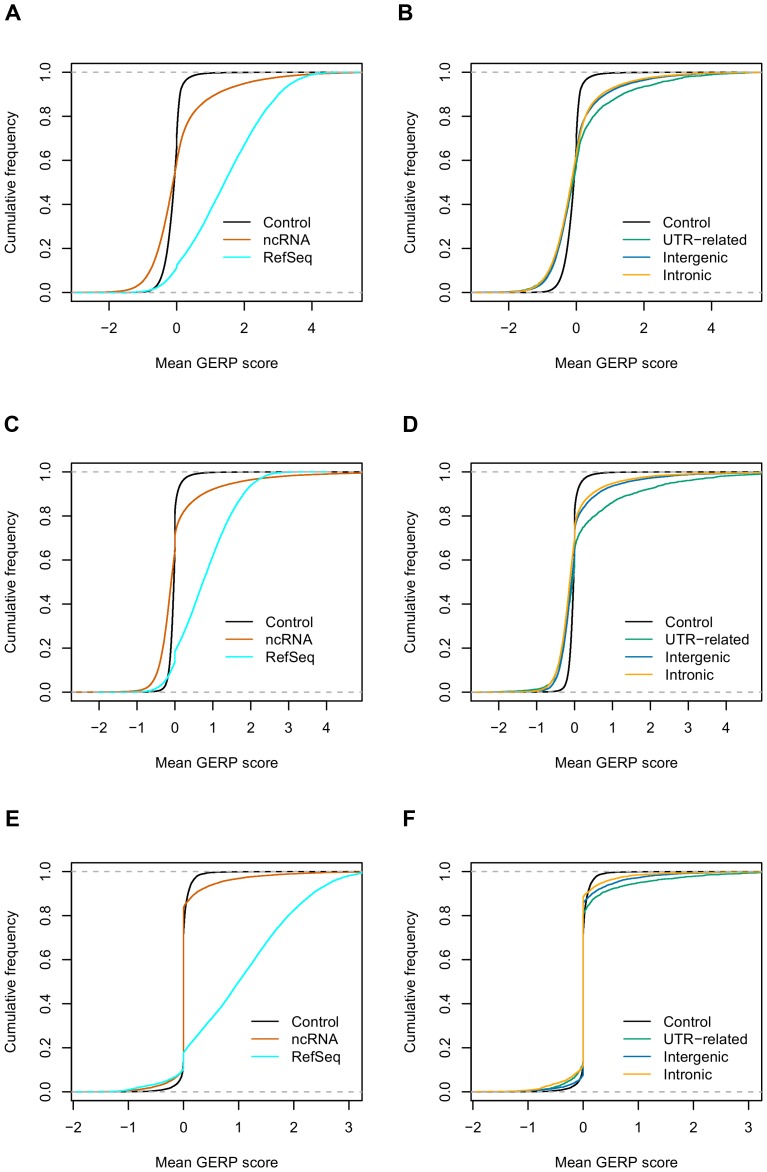
GERP++ score for ncRNAs identified from human, mouse and zebrafish. A and B are from human. C and D are from mouse. E and F are from zebrafish.

Most protein-coding genes are strongly conserved across different species, as judged by sequence alignment, and this characteristic is exploited to predict genes in newly sequenced organisms. However simple comparison of sequence alignment is insufficient to identify sequence conservation in ncRNAs because they are much less conserved than protein-coding genes. To analyze the evolutionary conservation of predicted ncRNAs, we used a maximum likelihood based method (GERP++ score) [33]. Overall, ncRNAs were conserved, compared to randomly selected un-transcribed genomic fragments, but they were less conserved than protein-coding genes (Figure 5). This result is consistent with previous observations [10], [25], [26], [34]. We also found that many ncRNAs (∼50% in human and ∼60% in mouse, based on GERP++ score) exhibited positive selection compared to control, randomly selected un-transcribed genomic regions (Figure 5A and 5C). Comparison of specific ncRNA subclasses showed that UTR-related RNAs were more conserved than intergenic ncRNAs, which in turn, were more conserved than intronic ncRNAs (Figure 5B, 5D and 5F). These observations were confirmed using two other methods, phastCons and phyloP (Figure S1 and Figure S2).

To compare the sequence conservation of our predicted ncRNAs with previously annotated long ncRNAs, we calculated the GERP++, phastCons and phyloP scores for human chromatin-based, enhancer-like and RNA-seq-based long ncRNAs (Figure S3, Figure S4 and Figure S5). Our predicted ncRNAs showed similar, but slightly more conserved cumulative conservation curves compared to all three known ncRNA datasets.

### 4 Intergenic ncRNAs are Preferentially Transcribed Proximate to Regulatory or Developmental Genes

Many ncRNAs, particularly intergenic ncRNAs can regulate gene transcription via different mechanisms [13], [20], [25], [35], including *cis*-regulatory mechanisms. We previously showed that intergenic ncRNAs were more likely to be close to regulatory genes [26]. We used the same methods to analyze the functional classification of human, mouse and zebrafish neighbor genes of gene-proximate intergenic ncRNAs. We chose intergenic ncRNAs located within 5 kb gene-flanking regions as “gene-proximate intergenic ncRNAs”, and used GO (Gene Ontology) to functionally classify these neighbor genes in human, mouse and zebrafish [36].

**Figure 6:**
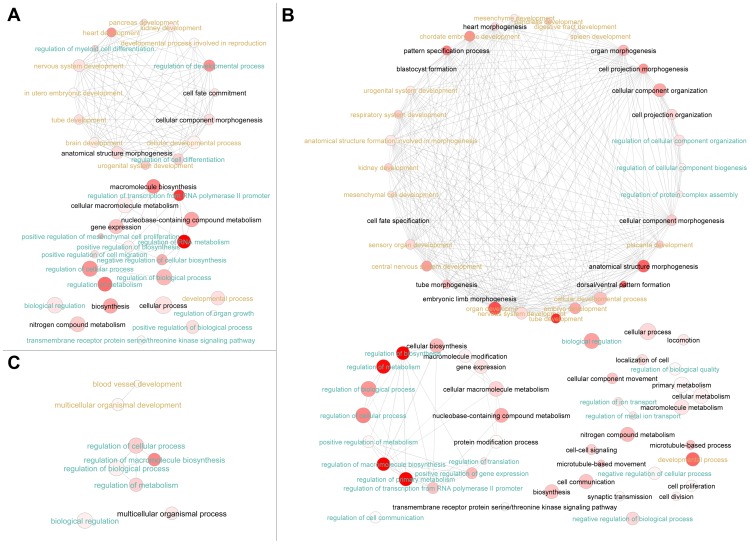
Over-represented GO terms of neighbor genes of 5′ end gene-proximate intergenic ncRNAs in human (A), mouse (B) and zebrafish (C). The bubble color indicates the P-value (EASE score from DAVID); bubble size indicates the frequency of the GO term in the underlying GOA database. Highly similar GO terms are linked by edges in the graph. Regulatory GO terms were highlighted with cyan-like colors, and developmental-associated GO terms were highlighted with gold colors.

**Figure 7:**
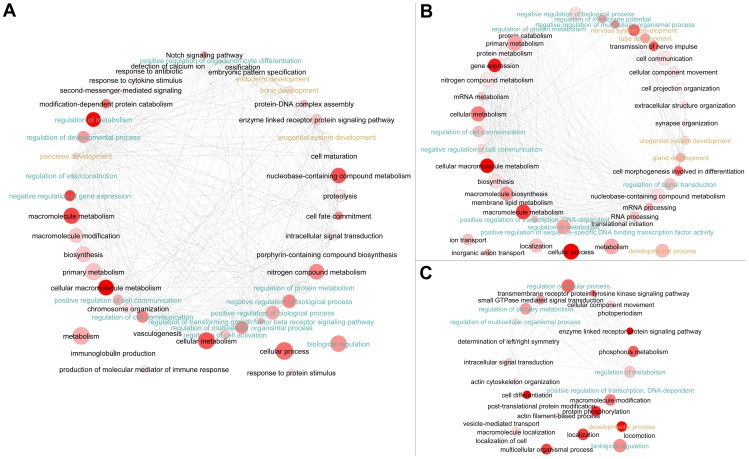
Over-represented GO terms of neighbor genes of 3′ end gene-proximate intergenic ncRNAs in human (A), mouse (B) and zebrafish (C). The bubble color indicates the P-value (EASE score from DAVID); bubble size indicates the frequency of the GO term in the underlying GOA database. Highly similar GO terms are linked by edges in the graph. Regulatory GO terms were highlighted with cyan-like colors, and developmental-associated GO terms were highlighted with gold colors.

**Figure 8:**
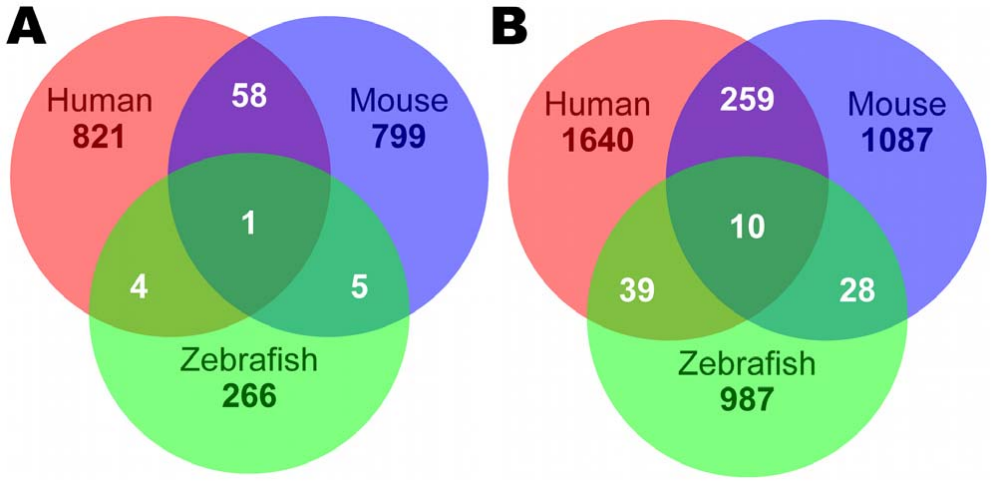
Venn diagrams show the conserved neighbor genes proximate to intergenic ncRNAs from human, mouse and zebrafish. A shows the intersection of neighbor genes with ncRNAs at their 5′ end. B shows the intersection of neighbor genes with ncRNAs at their 3′ end.

We found that genes with regulatory roles and/or associated with development were enriched in these neighbor genes across all three species with either 5′ end or 3′ end intergenic ncRNAs (Figure 6, Figure 7, Figure S6 and Figure S7). But very few of these neighbor genes were conserved across species, as confirmed by “Gene Symbol” comparison (Figure 8). However, 12 neighbor genes with 5′ proximate ncRNAs in human were found to have sequence-conserved correspondents in mouse and zebrafish neighbor genes, and 96 with 3′ proximate ncRNAs had sequence-conserved correspondents (Identity >60% and coverage >60%) (Table 4, Table S3). Significantly the vast majority of these neighbor genes with conserved proximate ncRNAs are expressed in human brain, suggesting a conserved *cis*-regulatory role for ncRNAs in brain gene expression. To determine if there was a biased functional distribution of protein-coding genes, many of which are 5 kb away from other protein-coding genes, we analyzed human GO annotation for all protein-coding genes with neighbor genes within 5 kb. We found no over-representation of regulatory or developmental genes in this set, indicating that a biased distribution of protein-coding genes did not affect our finding of enriched developmental and regulatory annotation for genes neighboring intergenic ncRNAs (Figure S8).

**Table 4:** Human genes conserved in mouse and zebrafish with proximate intergenic ncRNAs at their 5′ end (<5 kb).

Official_gene symbol	Expression inbrain (Human)*	Aliases & Descriptions	Diseases disorders*	Related ncRNAs
MAN1A1	Yes	Processing alpha-1,2-mannosidase IA | MAN9 |processing alpha-1,2-mannosidase IA | mannosyl-oligosaccharide 1,2-alpha-mannosidase IA |mannosidase, alpha, class 1A, member 1 | Man(9)-alpha-mannosidase | man(9)-alpha-mannosidase |Mannosidase alpha class 1A member 1 |HUMM3 |alpha-1,2-mannosidase IA | Alpha-1,2-mannosidase IA |Man9-mannosidase | HUMM9 |EC 3.2.1.113	Mannosidasedeficiency disease	N/A
MAN1A2	Yes	mannosidase, alpha, class 1A, member 2 |alpha-1,2-mannosidase IB | Mannosidase alpha class 1A member2 | mannosyl-oligosaccharide 1,2-alpha-mannosidase IB |alpha1,2-mannosidase | Processing alpha-1,2-mannosidase IB | processing alpha-1,2-mannosidase IB |MAN1B | Alpha-1,2-mannosidase IB |EC 3.2.1.113	N/A	N/A
ONECUT2	Yes	OC2 | hepatocyte nuclear factor 6-beta |ONECUT-2homeodomain transcription factor | HNF6B | One cuthomeobox 2 | HNF-6-beta | Hepatocyte nuclear factor6-beta | onecut 2 | OC-2 | one cut domain, familymember 2 | transcription factor ONECUT-2 | one cutdomain family member 2 | Transcription factorONECUT-2 | one cut homeobox 2	Oral cancer	Target of miR-9
PANK2	Yes	hPanK2 | pantothenate kinase 2 | FLJ11729 |neurodegeneration with brain iron accumulation 1(Hallervorden-Spatz syndrome) | NBIA1 |Hallervorden-Spatz syndrome | HARP | HSS | Pantothenic acid kinase2 | C20orf48 | pantothenic acid kinase 2 | PKAN |pantothenate kinase 2, mitochondrial |EC 2.7.1.33	Hallervorden-Spatz syndrome|dementia |dystonia	Host of miR-103
KCNJ4	Yes	IRK-3 | hIRK2 | IRK3 | inward rectifier K(+) channel Kir2.3| Potassium channel, inwardly rectifying subfamily Jmember 4 | HRK1 | HIRK2 | potassium channel, inwardlyrectifying subfamily J member 4 |hippocampal inwardrectifier potassium channel | potassium inwardly-rectifying channel, subfamily J, member 4 |Hippocampal inward rectifier | inward rectifier K+channel Kir2.3 | HIR | inward rectifier potassium channel4 | Kir2.3 | Inward rectifier K(+) channel Kir2.3	N/A	N/A
PDCD6IP	Yes	apoptosis-linked gene 2-interacting protein X |dopamine receptor interacting protein 4 | ALIX |programmed cell death 6 interacting protein | ALG-2-interacting protein 1 | programmed cell death 6-interacting protein | PDCD6-interacting protein | Hp95 |KIAA1375 | Alix | HP95 | AIP1 |ALG-2 interacting protein1 | DRIP4	N/A	Target ofmiR-1225-5P
SNX14	Yes	sorting nexin 14 | RGS-PX2 |sorting nexin-14	N/A	N/A
TUBB2B	Yes	tubulin beta-2B chain | tubulin, beta polypeptideparalog | MGC8685 | bA506K6.1 | tubulin, beta 2Bclass IIb | DKFZp566F223 | tubulin, beta 2B | classIIb beta-tubulin |class II beta-tubulin isotype	Lissencephaly	N/A
ZNF41	Yes	TUBB |class IIa beta-tubulin | tubulin, beta 2Aclass IIa | TUBB2 | tubulin, beta polypeptide 2 | tubulin,beta 2 | TUBB2B | dJ40E16.7 | tubulin beta-2A chain |tubulin, beta polypeptide | tubulin, beta 2A	Aland Island eye disease |mental disorder|intellectual disability	N/A
ZNF595	Yes	MRX89 |MGC8941 | zinc finger protein 41	N/A	N/A
ZNF676	Yes	FLJ31740 | zinc finger protein 595	N/A	N/A
ZNF761	No	zinc finger protein 676	N/A	N/A

* The expression and disease annotation were based on GeneCards V3 [57].

In order to determine if common GO terms were enriched across species, we compared all the significantly over-represented GO terms (p-value <0.05) across all three species. For genes with 5′ proximate intergenic ncRNAs, we found 19 over-represented terms in common, mostly concerning regulation of different biological pathways (Table 5). Specific molecular function terms enriched in all three species were “transcription factor activity” and “transcription regulator activity” (Table 5). In 3′ end neighbor genes, we found 34 significantly over-represented common GO terms, and the majority of them were “regulation” associated functional enrichments, also including “transcription factor activity” and “transcription regulator activity” (Table 6).

**Table 5:** GO terms in common from human, mouse and zebrafish neighbor genes within 5kb of proximate ncRNAs at their 5′ end.

Category	Term	*P value (human)	P value (mouse)	P value (zebrafish)
Molecular Function	GO:0003700∼transcription factoractivity	6.88E-07	0.001685935	0.002045234
Molecular Function	GO:0030528∼transcriptionregulator activity	2.80E-06	2.50E-05	0.001720193
Biological Process	GO:0006355∼regulation oftranscription, DNA-dependent	4.53E-06	0.000108619	0.02130028
Biological Process	GO:0051252∼regulation of RNAmetabolic process	7.91E-06	0.000178503	0.023870388
Biological Process	GO:0010556∼regulation ofmacromolecule biosyntheticprocess	8.37E-06	4.96E-07	0.000915362
Biological Process	GO:0060255∼regulation ofmacromolecule metabolicprocess	5.89E-05	7.41E-06	0.00691373
Biological Process	GO:0045449∼regulation oftranscription	6.20E-05	2.37E-06	0.001790827
Biological Process	GO:0031326∼regulation ofcellular biosynthetic process	8.41E-05	1.10E-06	0.001054761
Biological Process	GO:0009889∼regulation ofbiosynthetic process	0.000119902	1.33E-06	0.001088173
Biological Process	GO:0080090∼regulation ofprimary metabolic process	0.000146447	6.89E-07	0.002903755
Biological Process	GO:0010468∼regulation ofgene expression	0.000154686	1.42E-06	0.002943972
Biological Process	GO:0031323∼regulation ofcellular metabolic process	0.00015819	4.08E-06	0.002422663
Biological Process	GO:0019219∼regulation ofnucleobase, nucleoside,nucleotide and nucleic acidmetabolic process	0.000321532	7.14E-06	0.002751033
Biological Process	GO:0051171∼regulation ofnitrogen compound metabolicprocess	0.000343647	6.14E-06	0.002831208
Biological Process	GO:0019222∼regulation ofmetabolic process	0.000349372	1.09E-05	0.011044253
Biological Process	GO:0050794∼regulation ofcellular process	0.001348476	0.000766239	0.009737321
Biological Process	GO:0050789∼regulation ofbiological process	0.00433817	0.001382295	0.033481278
Biological Process	GO:0065007∼biologicalregulation	0.022428992	0.002031998	0.031603795
Biological Process	GO:0007275∼multicellularorganismal development	0.035916788	0.000243142	0.043621824

* The GO terms were ordered by p-value in human.

**Table 6:** GO terms in common from human, mouse and zebrafish neighbor genes within 5kb of proximate ncRNAs at their 3′ end.

Category	Term	*P value (human)	P value (mouse)	P value (zebrafish)
Molecular Function	GO:0003677∼DNA binding	2.52E-07	0.001016369	0.022517442
Biological Process	GO:0019222∼regulation of metabolic process	5.94E-06	0.001833053	0.007240134
Biological Process	GO:0031323∼regulation of cellular metabolic process	7.06E-06	0.001932015	0.002531781
Biological Process	GO:0080090∼regulation of primary metabolic process	8.71E-06	0.000746433	0.001635905
Biological Process	GO:0060255∼regulation of macromoleculemetabolic process	1.52E-05	0.001021052	0.015088588
Cellular Component	GO:0044464∼cell part	2.64E-05	0.005138983	0.021192768
Cellular Component	GO:0005623∼cell	2.75E-05	0.005138983	0.021192768
Biological Process	GO:0009889∼regulation of biosynthetic process	4.64E-05	0.00153235	0.001998668
Biological Process	GO:0010556∼regulation of macromolecule biosynthetic process	5.07E-05	0.001133669	0.004636373
Biological Process	GO:0031326∼regulation of cellular biosynthetic process	5.93E-05	0.001770385	0.002769539
Biological Process	GO:0010468∼regulation of gene expression	6.05E-05	0.001153647	0.019089475
Biological Process	GO:0019219∼regulation of nucleobase, nucleoside,nucleotide and nucleic acid metabolic process	7.45E-05	0.002835006	0.006403442
Biological Process	GO:0045449∼regulation of transcription	9.02E-05	0.001133423	0.009147674
Biological Process	GO:0051171∼regulation of nitrogen compound metabolic process	0.000115522	0.003953563	0.006560818
Molecular Function	GO:0003700∼transcription factor activity	0.000701959	0.006403948	0.003113804
Biological Process	GO:0051252∼regulation of RNA metabolic process	0.002751656	0.012593576	0.006423226
Biological Process	GO:0006355∼regulation of transcription, DNA-dependent	0.002836401	0.008313995	0.007792617
Molecular Function	GO:0030528∼transcription regulator activity	0.003105196	0.00782068	0.001014153
Biological Process	GO:0031328∼positive regulation of cellular biosynthetic process	0.007428451	0.007226598	0.033533698
Biological Process	GO:0009891∼positive regulation of biosynthetic process	0.007469104	0.008740921	0.033533698
Biological Process	GO:0010557∼positive regulation of macromolecule biosynthetic process	0.009196945	0.003489005	0.028269774
Biological Process	GO:0010628∼positive regulation of gene expression	0.010415711	0.009098997	0.021490484
Biological Process	GO:0045941∼positive regulation of transcription	0.011143783	0.00569233	0.021490484
Molecular Function	GO:0005515∼protein binding	0.017163574	0.000809527	1.60E-06
Biological Process	GO:0045893∼positive regulation of transcription, DNA-dependent	0.02105859	0.004978895	0.012497621
Molecular Function	GO:0008270∼zinc ion binding	0.022962024	0.003010259	0.036242576
Biological Process	GO:0048869∼cellular developmental process	0.024154786	0.006314016	9.66E-07
Biological Process	GO:0051254∼positive regulation of RNA metabolic process	0.024566919	0.005669422	0.014428949
Biological Process	GO:0030154∼cell differentiation	0.02953709	0.007655265	1.65E-06
Biological Process	GO:0045935∼positive regulation of nucleobase, nucleoside, nucleotide and nucleic acid metabolic process	0.03326329	0.011738803	0.039427105
Biological Process	GO:0048468∼cell development	0.033319932	0.007737614	0.003006631
Biological Process	GO:0051173∼positive regulation of nitrogen compound metabolic process	0.033319932	0.012196797	0.04261773
Biological Process	GO:0044267∼cellular protein metabolic process	0.042639534	0.003735008	0.011732507
Biological Process	GO:0001655∼urogenital system development	0.048304941	0.012438853	0.04591464

* The GO terms were ordered by p-value in human.

Taken together, these results indicated that many intergenic ncRNAs were transcribed proximate to regulatory or developmental genes in human, mouse and zebrafish. This positional bias and functional classification of neighbor genes indicated a potential *cis*-regulatory role for intergenic ncRNAs in the transcription of protein-coding genes.

## Discussion

We have assembled and annotated the non-protein-coding transcriptome from human, mouse and zebrafish in a stringent and comprehensive fashion using all publically available ESTs. Our results increase the number of annotated ncRNAs by more than an order of magnitude and are robust and highly significant for the following reasons. First, ESTs used to assemble long ncRNAs were generated from multiple libraries from a broad spectrum of tissues/cell types, developmental stages or biological circumstances. Second, robust, highly stringent selection procedures used to assemble long ncRNAs enabled us to remove possible sequencing artifacts. Third, ESTs generated by traditional sanger sequencing technology gave longer raw reads and could be assembled into longer and more accurate consensus transcripts than possible with short read sequencing technologies used in previous studies [12], [14], [24]. In spite of these positive attributes we also have to acknowledge the potential shortcomings of our reconstructed long ncRNAs. First, many ESTs were archived without transcription orientation, thus it was difficult to deduce transcription orientations for some reconstructed ncRNAs. Second, reconstruction of ESTs from different libraries might have resulted in loss of alternative transcripts. Third, although longer raw reads enabled us to build long consensus transcripts with high accuracy, many reconstructed transcripts are possibly still not full-length. One limitation of our results stemmed from our decision to specifically exclude repetitive ESTs from our analysis because they confounded our sequence reconstructions. This means that repeat containing ncRNAs were not included in our results.

Intergenic ncRNAs from all three species showed the same positional bias in their distribution with respect to protein-coding genes, consistent with previous observations in cow [26]. Because this positional bias was also previously reported in long intergenic ncRNAs identified using quite different methods [27], [30], [31], [37], we propose that this is a common property for intergenic ncRNAs across vertebrate species. This biased genomic distribution could result from two possible scenarios: First, the observed positional bias is a functional attribute for intergenic ncRNAs because they *cis-*regulate nearby protein-coding genes through a number of possible mechanisms. Many long intergenic ncRNAs, such as enhancer-like ncRNAs and promoter-associated ncRNAs, have been validated as *cis*-regulators of nearby protein-coding genes [25], [38], [39]. The transcription of these long intergenic ncRNAs may remodel the chromatin status of surrounding regions, including the promoters of protein-coding loci [18], [40], [41], [42]. Another possibility is that transcription of long ncRNAs from promoter regions of protein-coding genes competes for the transcription-binding complex between long ncRNAs and nearby genes, thus balancing their transcription [17], [43], [44]. Although many long ncRNAs have been experimentally validated and fed into different gene regulation models, more functional manipulations of long ncRNAs are required to test different regulatory models. The second scenario is that these ncRNAs are fragments of un-annotated UTRs or alternative splicing isoforms. Current ncRNA identification methods are heavily reliant on the available gene models, which may be incomplete. This possibility has some support because some gene-proximate intergenic ncRNAs were similar to UTRs. Because of this possibility, all functional classifications in our analysis were based on stringent intergenic ncRNAs (all UTR-related RNAs removed). However we also observed a large number of antisense transcripts within the gene-proximate intergenic ncRNAs, which cannot be categorized as possible UTRs. Moreover, many studies have identified pervasive, independent functional non-coding transcripts from gene-proximate regions, even in UTRs of protein-coding genes [32]. We conclude that our gene-proximate intergenic ncRNAs are most likely functional, but that we need to wait for further experimental testing to understand how they work [45]. We put forward our ncRNAs as good starting points for functional screening.

Long ncRNAs are pervasively transcribed across genomes in different species [1], [46], [47]. However, the true number of long ncRNAs is still not known. Previous studies using whole-genome tiling arrays demonstrated that the majority of the human genome was transcribed [2], [3], [48]. The FANTOM project also revealed thousands of long ncRNAs based on cDNAs in mouse [6]. In the past few years, different categories of long ncRNAs, particularly lincRNAs, have been annotated using a variety of methods [10], [11], [12], [14], [24], [25]. Our ncRNAs are novel because we screened out ESTs with significant similarity to RefSeqs (coding and non-coding). This novelty is confirmed by the limited overlap of our ncRNAs with previous ncRNAs. In order to assess our methodology vis a vis previous methods, we aligned previously reported ncRNAs against the raw EST data we used as input for our pipeline (See Material S1). Generally ncRNAs from other datasets based on transcriptome data were present in the ESTs, but this was not the case with ncRNAs based on prediction from chromatin state [10], [11]. When we assessed the expression of previously reported ncRNAs from chromatin state [10], [11] we found that many of these predicted ncRNAs showed no evidence of transcription based on ESTs. These ncRNAs were validated by using tiling array based expression analysis with reported expression levels of 70% within single tissues/cell types [11]. Because we found no more than 46% of these in the raw human EST data (Figure 4, Table S2 and Material S1), we re-visited the tiling arrays reported for the validation. Most of the chromatin state based predicted ncRNAs contained repeats and about 38% of the tiling array probes used to validate them also contained repetitive sequence (Material S1). It is likely that the reported tiling array validation of 70% of the chromatin state predicted ncRNAs is an inflated estimate, as many transcripts contain repeats in their UTRs which would cross-hybridize to these probes, providing false positive signals. On the whole, the number of ncRNAs that were not found in ESTs was a tiny fraction of the total number of ncRNAs included in previous publications and in the present report. We conclude that the number of ncRNAs, particularly for intergenic, repeat containing ncRNAs, is significantly underestimated based on our current knowledge.

Sequence conservation is an important functional signature of genomic transcripts. Many of the ncRNAs that we identified, even though they are clearly less conserved than protein-coding genes, show clear sequence conservation compared to randomly selected, un-transcribed genomic fragments. Furthermore, intergenic ncRNAs are more conserved than intronic ncRNAs in all three species. This weak but significant purifying selection of lincRNAs was observed in a previous study [49] and these results are also consistent with the conservation levels of ncRNAs previously identified from cow [26], as well as previously reported long ncRNA datasets [10], [12], [14].

Sequence conservation is not the only benchmark for functional significance, as we also observed a small number of protein-coding genes under positive selection. Genes for ncRNAs probably evolve more rapidly than protein-coding genes, which are constrained by triplet codons to maintain the conserved functions of translated proteins. For functional ncRNAs, such as microRNAs, conserved secondary structures have been identified as functional elements required to regulate gene expression. Conserved secondary structures may be more important than conserved primary sequence for long ncRNAs [34]. Furthermore, because many long ncRNAs are transcribed in tissue/cell-type specific fashion [12], [14], [24], [50], [51] we suggest that many ncRNAs might be species-specific. The overall lack of correspondence between neighbor genes with proximate intergenic ncRNAs across species supports the idea that ncRNAs evolve rapidly, generating species-specific patterns of tissue specific, developmental regulation. ncRNAs undergoing positive selection might represent novel tissue/cell-type/species specific regulatory transcripts. A significant exception to the lack of correspondence between neighbor genes and proximate intergenic ncRNAs was the conservation of 108 genes with proximate ncRNAs in human, mouse and zebrafish. 97 of these genes are expressed in human brain, suggesting a conserved *cis*-regulatory role for ncRNAs in brain development. Previously, Chodroff *et al.* [52] showed that four conserved long ncRNAs also had conserved expression in brain across a range of amniotes. Our results indicate that conservation of ncRNA association with protein-coding genes expressed in brain also occurs (Table 4, Table S3), suggesting the vertebrates possess a conserved co-expression or *cis-*regulatory network of ncRNA/gene pairs.

As discussed above, the biased positional distribution of intergenic ncRNAs suggested *cis*-regulatory functions. The functional annotation of neighbor genes with nearby intergenic ncRNAs supports this hypothesis. Many intergenic ncRNAs are preferentially transcribed from regions adjacent to regulatory and developmental genes as seen in this report and on a smaller scale by others [10], [24], [38].

In conclusion, we present a significantly expanded set of ncRNAs that suggests that ncRNAs, while exhibiting sequence conservation, evolve rapidly in terms of their association with neighboring regulatory and developmental genes. The exception to this rapid evolution appears to be with respect to a subset of genes expressed in brain. Long ncRNAs, such as intergenic ncRNAs, may function through different mechanisms as genome wide regulatory elements in many biological pathways, including brain development [53].

## Methods

### 1 ncRNA Identification from Human, Mouse and Zebrafish

ncRNA identification was performed using a previously built pipeline [26]. First, all available ESTs were extracted from dbEST (NCBI). After removing low quality sequences and ESTs composed mostly of repetitive elements, all remaining ESTs were clustered and assembled into longer unique consensus transcripts. Protein-coding genes were removed from the unique transcripts based on similarity searches against RefSeqs and Swiss-Prot databases. As a final step, transcripts were checked for ORFs to remove potential un-annotated protein-coding genes. This left a set of long ncRNAs. To further reduce the redundancy of these long ncRNAs, we reconstructed all putative long ncRNAs based on their genomic coordinates using inchworm [54].

The classification of ncRNAs into three different categories, intronic, intergenic and overlapped ncRNAs with respect to protein-coding genes was performed with R as previously described [26]. The intergenic ncRNAs that were located within 1 kb of the 5′ and 3′ ends of protein-coding genes, or with sequence similarity against known UTRs, were further classified as UTR-related RNAs. All remaining intergenic ncRNAs were classified as *bona fide* intergenic ncRNAs.

### 2 Neighbor Genes and Transcription Orientation of ncRNAs with Respect to Neighbor Genes

The closest protein-coding gene to an intergenic ncRNA was chosen as the neighbor gene of this intergenic ncRNA. The transcriptional orientation of ncRNAs was determined based on two criteria: First, many ESTs extracted from NCBI have cloning and sequencing information, which was used to determine the transcription orientation of both singletons and contigs. Second, the transcription orientation of spliced long ncRNAs was deduced from splicing information when they were mapped onto the genome. The “sense” intergenic ncRNAs were defined as transcribing from the same strand as neighbor genes, and *vice versa*.

### 3 Comparisons with Known Well-characterized Long ncRNAs in Human, Mouse and Zebrafish

The sources and summary information for previously characterized ncRNAs are shown in Table 7. For chromatin-based lincRNAs in human and mouse, we used the exons instead of the long chromatin regions as the known lincRNAs. The overlap of our EST-based ncRNAs with these known long ncRNA datasets were analyzed with the “GenomicFeatures” R package.

**Table 7:** Previously annotated long ncRNA datasets used for comparison.

Dataset	Number of ncRNAs	Source	Method	Reference
Chromatin-based lincRNAs (Human)	4,860*	10 cell types	Chromatin signatureidentification (K4–K36 domain)	Khalil AM, 2009 [11]
Enhancer-like long ncRNAs (Human)	3,011	Multiple	Screening from GENCODEannotation	Orom UA, 2010 [25]
RNA-seq-based lincRNAs (Human)	8,195	24 tissues and cell types	Screening from assembledRNA-seq data	Cabili MN, 2011 [24]
Chromatin-based lincRNAs (Mouse)	2,127*	4 cell types	Chromatin signatureidentification (K4–K36 domain)	Guttman M, 2009 [10]
RNA-seq-based lincRNAs (Mouse)	1,140	3 cell types	Screening from assembledRNA-seq data	Guttman M, 2010 [12]
RNA-seq-based long ncRNAs (Zebrafish)	1,133	8 embryonic stages	Screening from assembledRNA-seq data	Pauli A, 2011 [14]

* These are the exons identified by microarray from non-coding k4-k36 domains.

### 4 Conservation Analyses of ncRNAs

Three different conservation scores were used to analyze the sequence conservation of ncRNAs. The GERP++ scores for human and mouse were downloaded from http://mendel.stanford.edu/SidowLab/downloads/gerp/. For zebrafish, the GERP++ scores were calculated with GERP++ tool based on the multiple alignments of 7 genomes (hg19/GRCh37, mm9, xenTro2, tetNig2, fr2, gasAcu1, oryLat2) with danRer7 of zebrafish. The phastCons scores and phyloP scores for human, mouse and zebrafish were downloaded from UCSC based on genome assembly hg19/GRCh37 (human), mm9 (mouse) and danRer7 (zebrafish) respectively. The mean GERP++/phastCons/phyloP score for each ncRNA/RefSeq/control sequence was calculated by normalizing the sum of GERP++/phastCons/phyloP scores against the length of the sequence. All RefSeqs excluding “NR” and “XR” entries (non-coding transcripts) were used as the protein-coding gene dataset. The same number of genomic fragments as ncRNAs, which ranged in size from 500 bp to 15,000 bp, were randomly selected from un-transcribed genomic regions (no ESTs mapped) as the control datasets for each species respectively. The cumulative frequency for each dataset was calculated and plotted using the R package.

### 5 Functional Classifications of Neighbor Genes of Gene-proximate Intergenic ncRNAs

Gene-proximate intergenic ncRNAs were selected from stringent intergenic ncRNAs located within 5 kb of the 5′ and 3′ ends of protein-coding genes. GO classification of neighbor genes was performed on the DAVID (Database for Annotation, Visualization and Integrated Discovery) web server [55]. The thresholds for over-represented GO terms were set as gene count >5 and p-value (EASE score) <0.05. The web server REViGO was used to reduce the redundancy and visualize the overrepresented GO terms based on semantic similarity [56].

The gene symbols of neighbor genes with annotations in GO were compared across species to find common genes. BLAST was used to carry out sequence similarity searches for conserved neighbor genes across all three species.

All protein-coding genes with neighbor genes located in their 5 kb flanking regions were analysed in the same fashion as neighbor genes of intergenic ncRNAs.

## Supporting Information

Figure S1 **PhastCons scores of ncRNAs identified from human (A, B), mouse (C, D) and zebrafish (E, F).**
(TIF)

Figure S2 **Phylop Scores of identified ncRNAs from human (A, B), mouse (C, D) and zebrafish (E, F).**
(TIF)

Figure S3 **Comparison of GERP++ scores of our ncRNAs with previously published lincRNA datsets in human.**
(TIF)

Figure S4 **Comparison of phastCons scores of our ncRNAs with previously published human lincRNA datasets.**
(TIF)

Figure S5 **Comparison of phyloP scores of our ncRNAs with previously published human lincRNA datasets.**
(TIF)

Figure S6 **The “Treemap” view of over-represented GO terms of neighbor genes with 5′ end gene-proximate intergenic ncRNAs in human (A), mouse (B) and zebrafish (C).** Each rectangle represents a single cluster. The clusters are joined into ‘superclusters’ of loosely related terms, visualized with different colors. The size of the rectangles was adjusted to reflect the P-value (EASE score in DAVID) of the GO term, with a larger rectangle corresponding to a smaller p-value.
(TIF)

Figure S7 **The “Treemap” view of over-represented GO terms of neighbor genes with 3′ end gene-proximate intergenic ncRNAs in human (A), mouse (B) and zebrafish (C).** Each rectangle represents a single cluster. The clusters are joined into ‘superclusters’ of loosely related terms, visualized with different colors. The size of the rectangles was adjusted to reflect the P-value (EASE score in DAVID) of the GO term, with a larger rectangle corresponding to a smaller p-value.
(TIF)

Figure S8 **Over-represented GO terms for all protein-coding genes with neighbor genes within 5 kb in human.**
(TIF)

Table S1 **Genomic coordinates of predicted ncRNAs in human, mouse and zebrafish.** This excel file contains genomic coordinates of predicted ncRNAs identified by our pipeline in human (sheet 1), mouse (sheet 2) and zebrafish (sheet 3).
(XLSX)

Table S2 **Summary of human and mouse known long ncRNAs that align to ESTs.** This table contains a summary of human known long ncRNAs (chromatin-based, enhancer-like and RNA-seq based) and mouse long ncRNAs (chromatin-based, RNA-seq based) mapped against ESTs.
(DOCX)

Table S3 **Annotation of common protein-coding genes with proximate intergenic ncRNAs (<5 kb) in human, mouse and zebrafish.** Sheet 1 in this excel table shows 12 conserved genes with ncRNAs at the 5′ end and sheet 2 shows 96 conserved genes with ncRNAs at the 3′ end.
(XLSX)

Material S1 **Supporting results.**
(DOCX)
