# Supplementary figures and images for: Identification and Comparative Analysis of ncRNAs in Human, Mouse and Zebrafish Indicate a Conserved Role in Regulation of Genes Expressed in Brain
Source: PLoS One. 2012 Dec 20;7(12):e52275. doi: 10.1371/journal.pone.0052275 (PMC3527520; doi:10.1371/journal.pone.0052275)

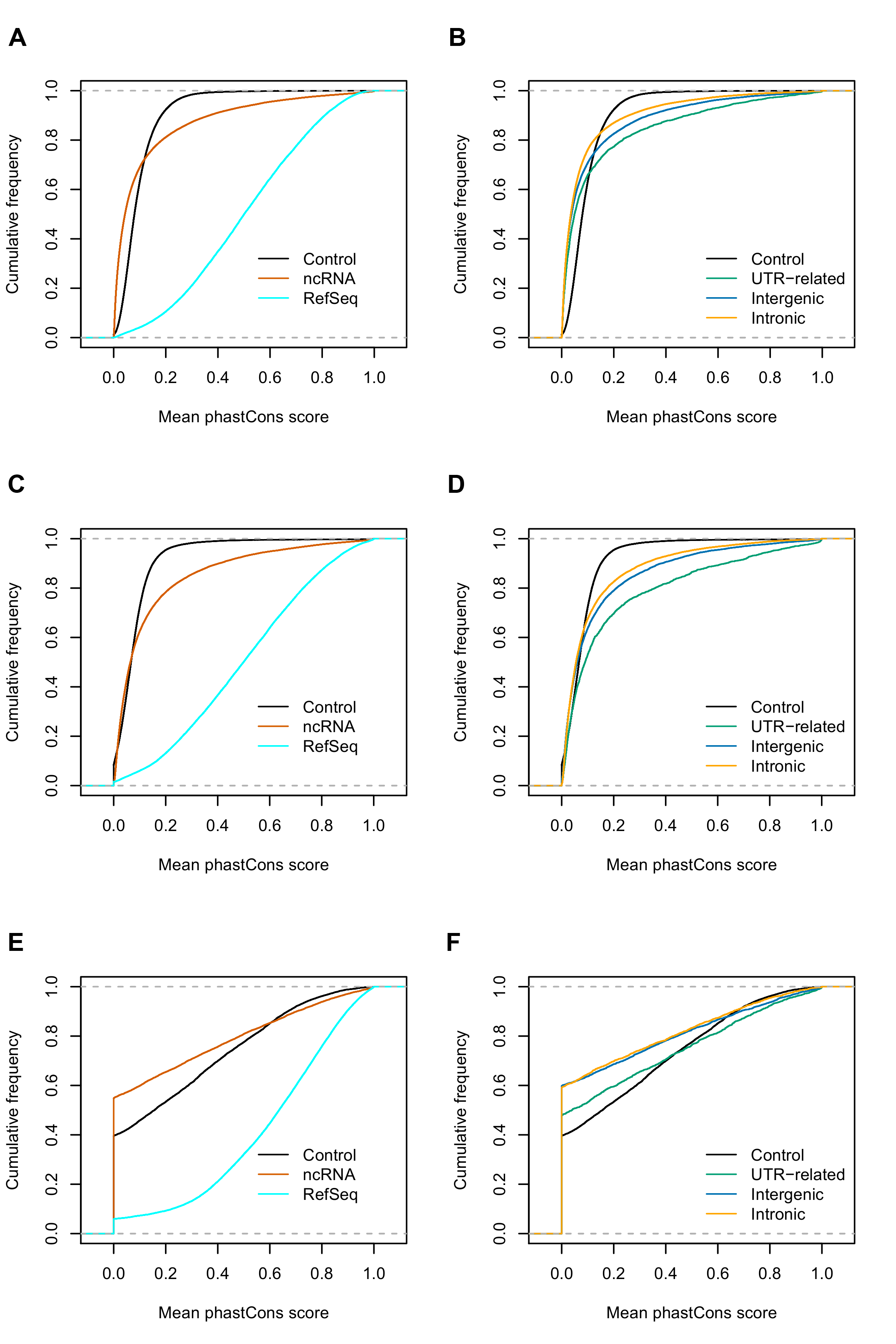

Supplement: Figure S1 — PhastCons scores of ncRNAs identified from human (A, B), mouse (C, D) and zebrafish (E, F). (TIF) [file pone.0052275.s001.tif]

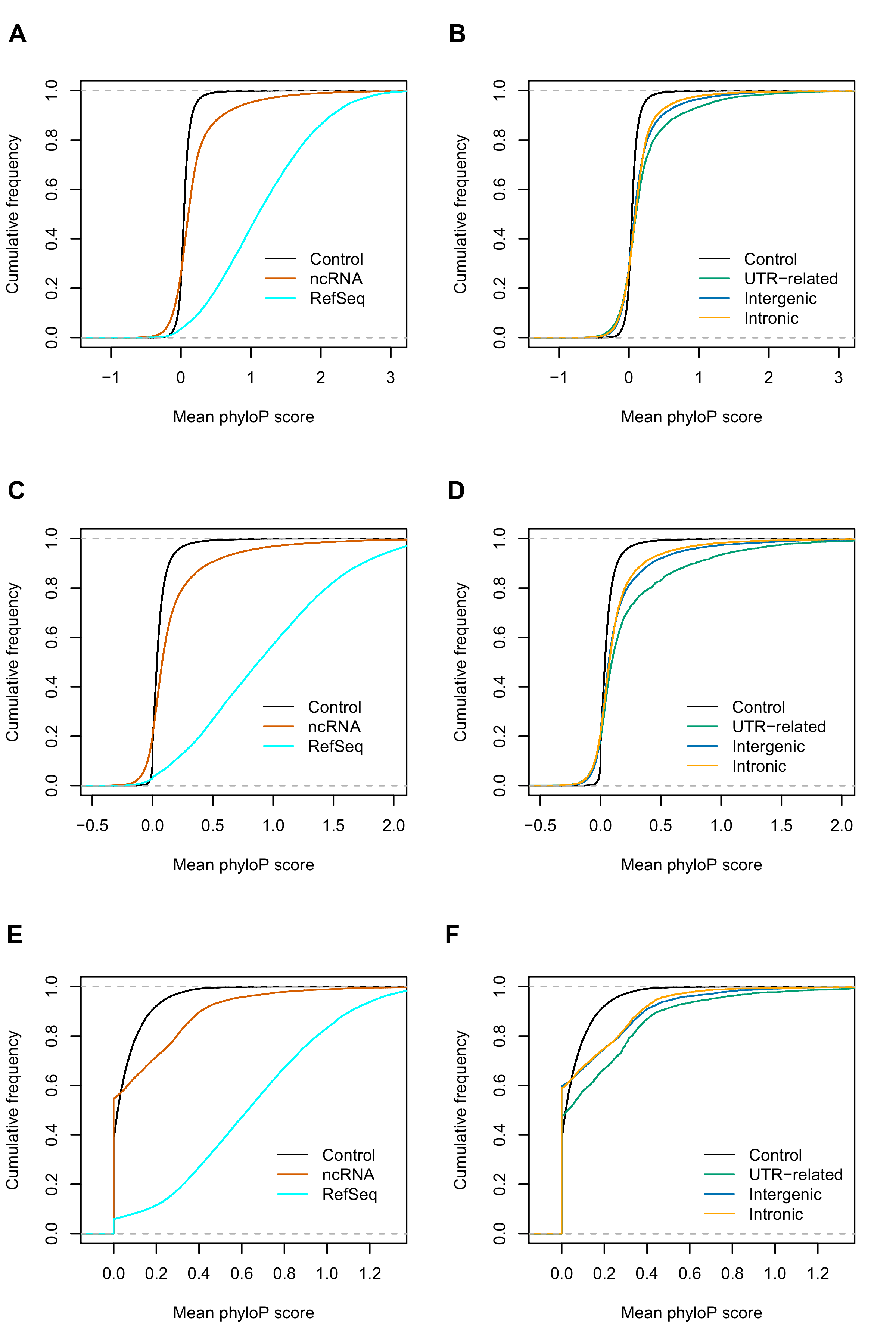

Supplement: Figure S2 — Phylop Scores of identified ncRNAs from human (A, B), mouse (C, D) and zebrafish (E, F). (TIF) [file pone.0052275.s002.tif]

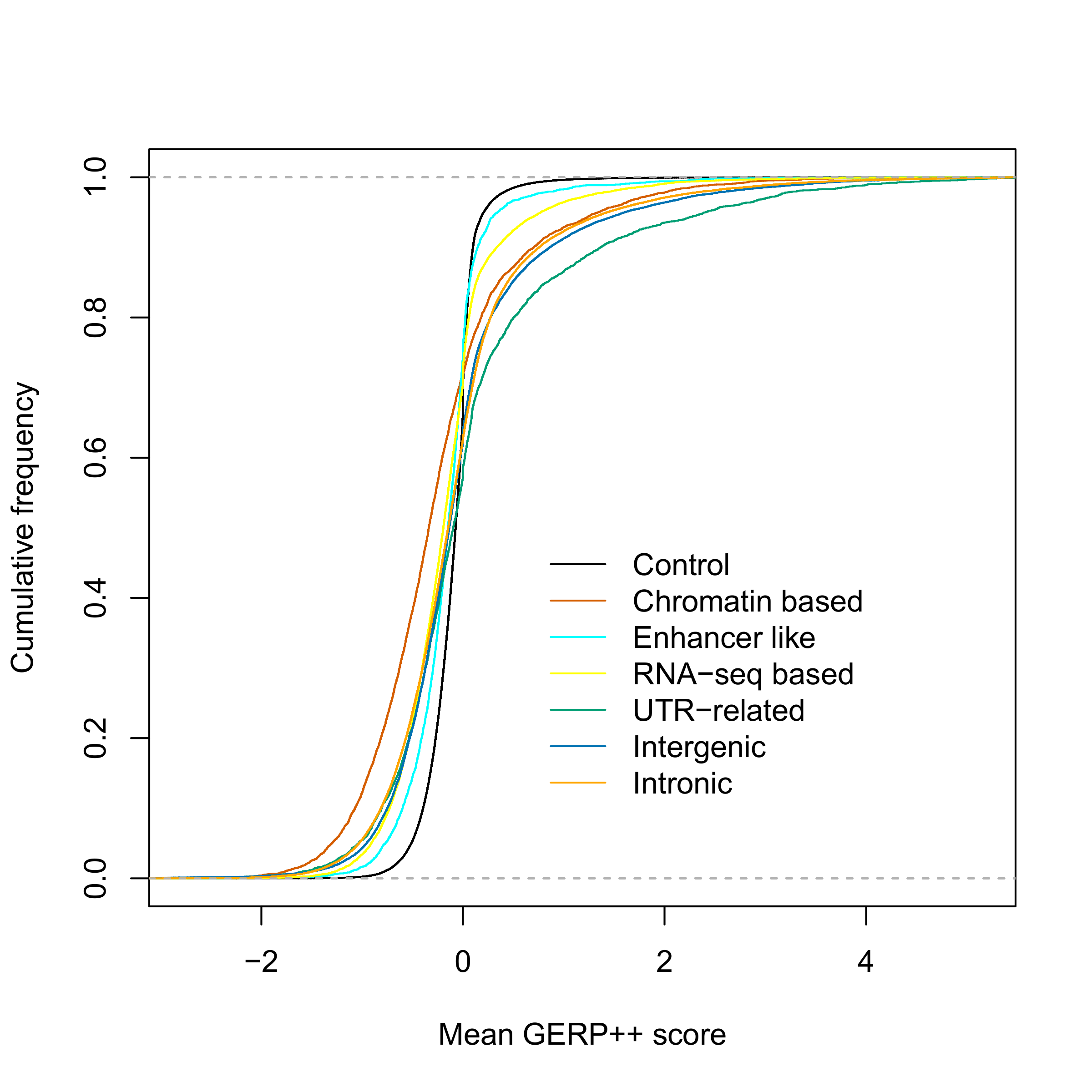

Supplement: Figure S3 — Comparison of GERP++ scores of our ncRNAs with previously published lincRNA datsets in human. (TIF) [file pone.0052275.s003.tif]

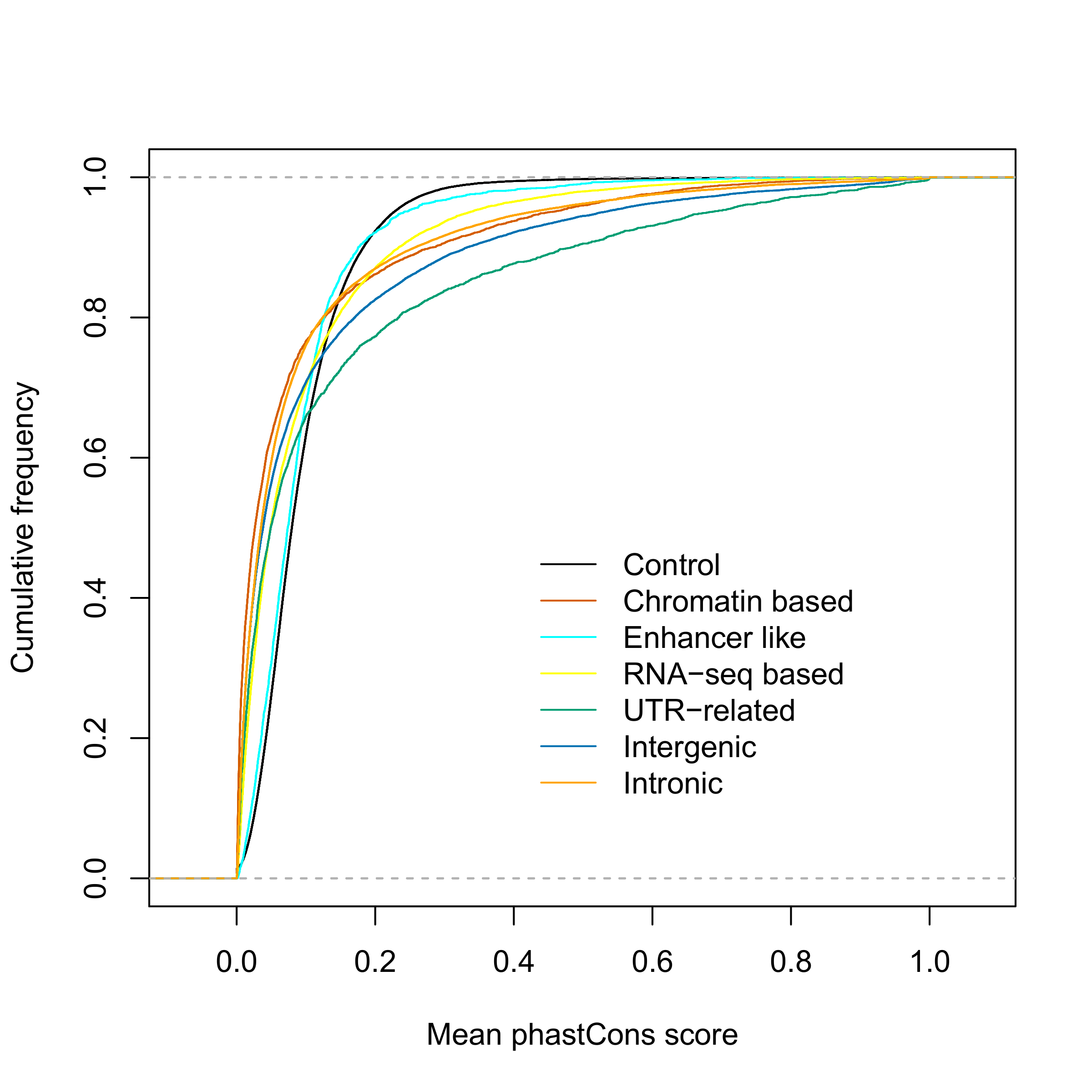

Supplement: Figure S4 — Comparison of phastCons scores of our ncRNAs with previously published human lincRNA datasets. (TIF) [file pone.0052275.s004.tif]

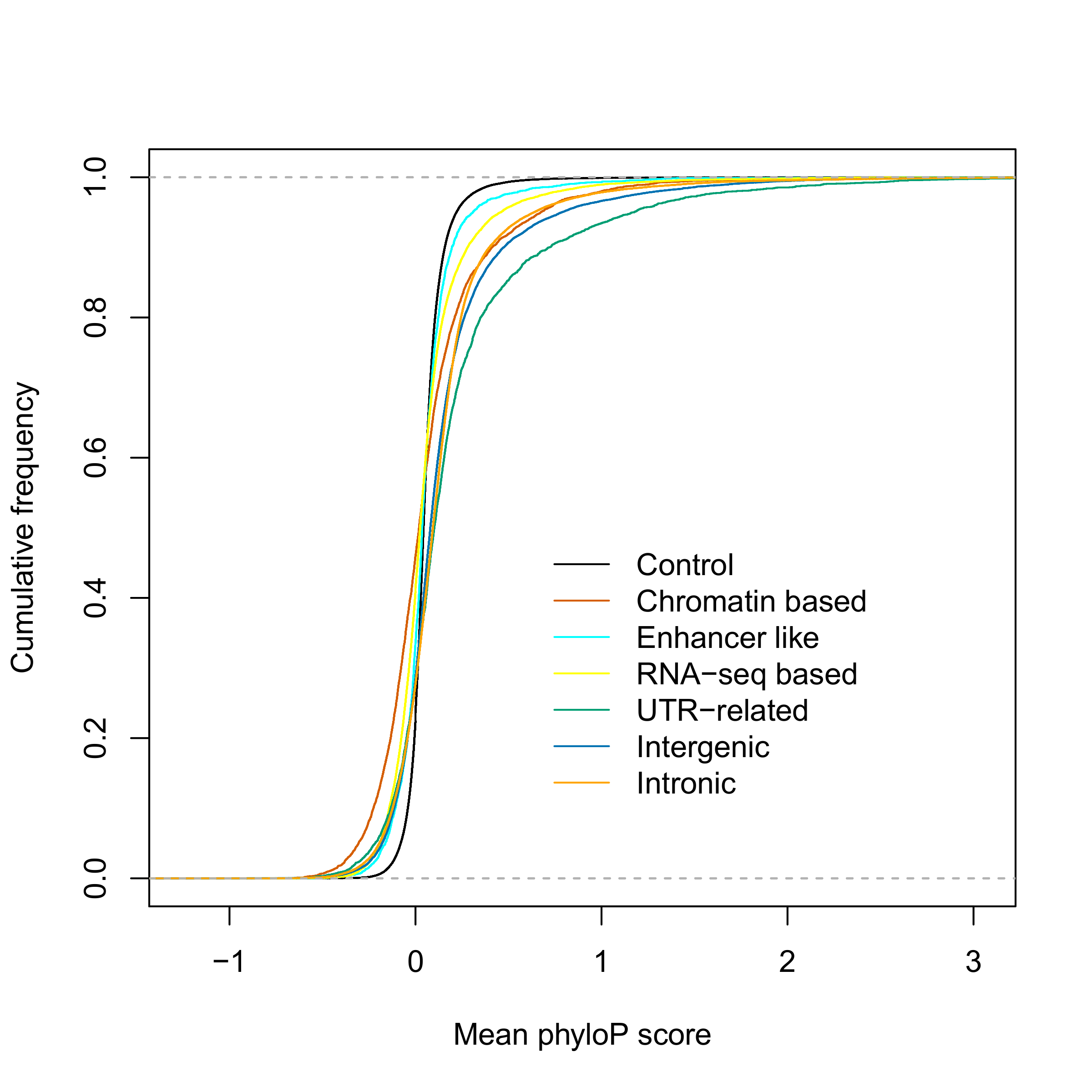

Supplement: Figure S5 — Comparison of phyloP scores of our ncRNAs with previously published human lincRNA datasets. (TIF) [file pone.0052275.s005.tif]

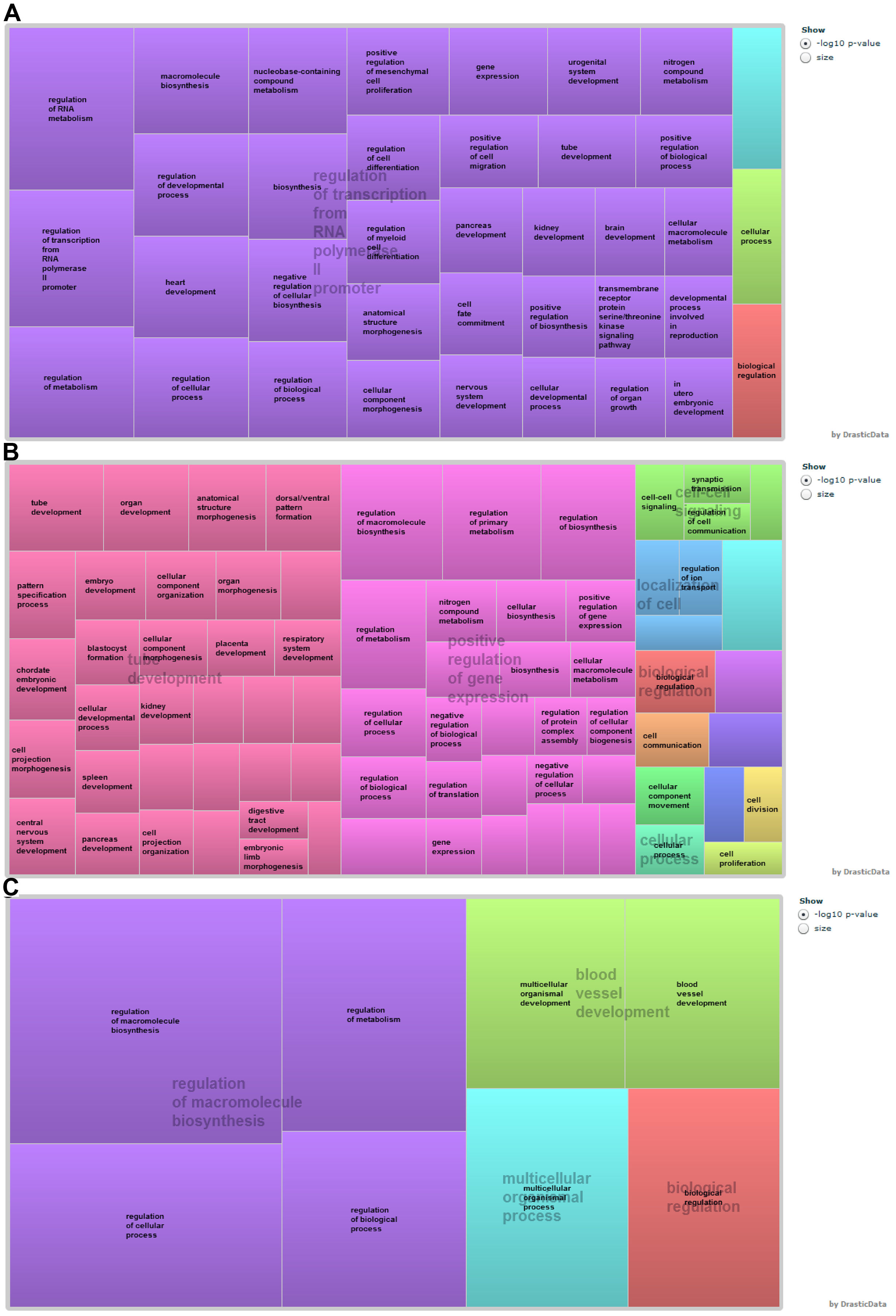

Supplement: Figure S6 — The “Treemap” view of over-represented GO terms of neighbor genes with 5′ end gene-proximate intergenic ncRNAs in human (A), mouse (B) and zebrafish (C). Each rectangle represents a single cluster. The clusters are joined into ‘superclusters’ of loosely related terms, visualized with different colors. The size of the rectangles was adjusted to reflect the P-value (EASE score in DAVID) of the GO term, with a larger rectangle corresponding to a smaller p-value. (TIF) [file pone.0052275.s006.tif]

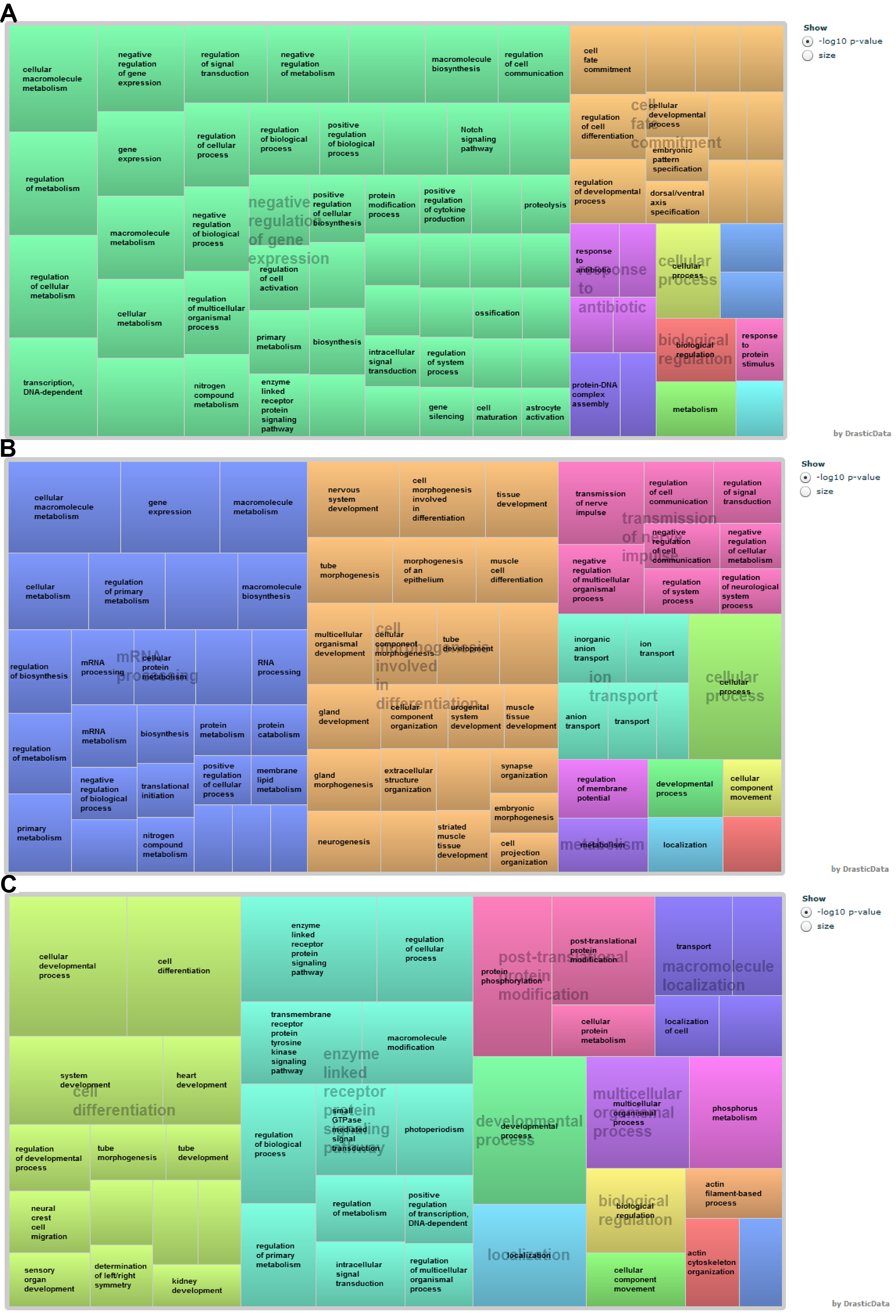

Supplement: Figure S7 — The “Treemap” view of over-represented GO terms of neighbor genes with 3′ end gene-proximate intergenic ncRNAs in human (A), mouse (B) and zebrafish (C). Each rectangle represents a single cluster. The clusters are joined into ‘superclusters’ of loosely related terms, visualized with different colors. The size of the rectangles was adjusted to reflect the P-value (EASE score in DAVID) of the GO term, with a larger rectangle corresponding to a smaller p-value. (TIF) [file pone.0052275.s007.tif]

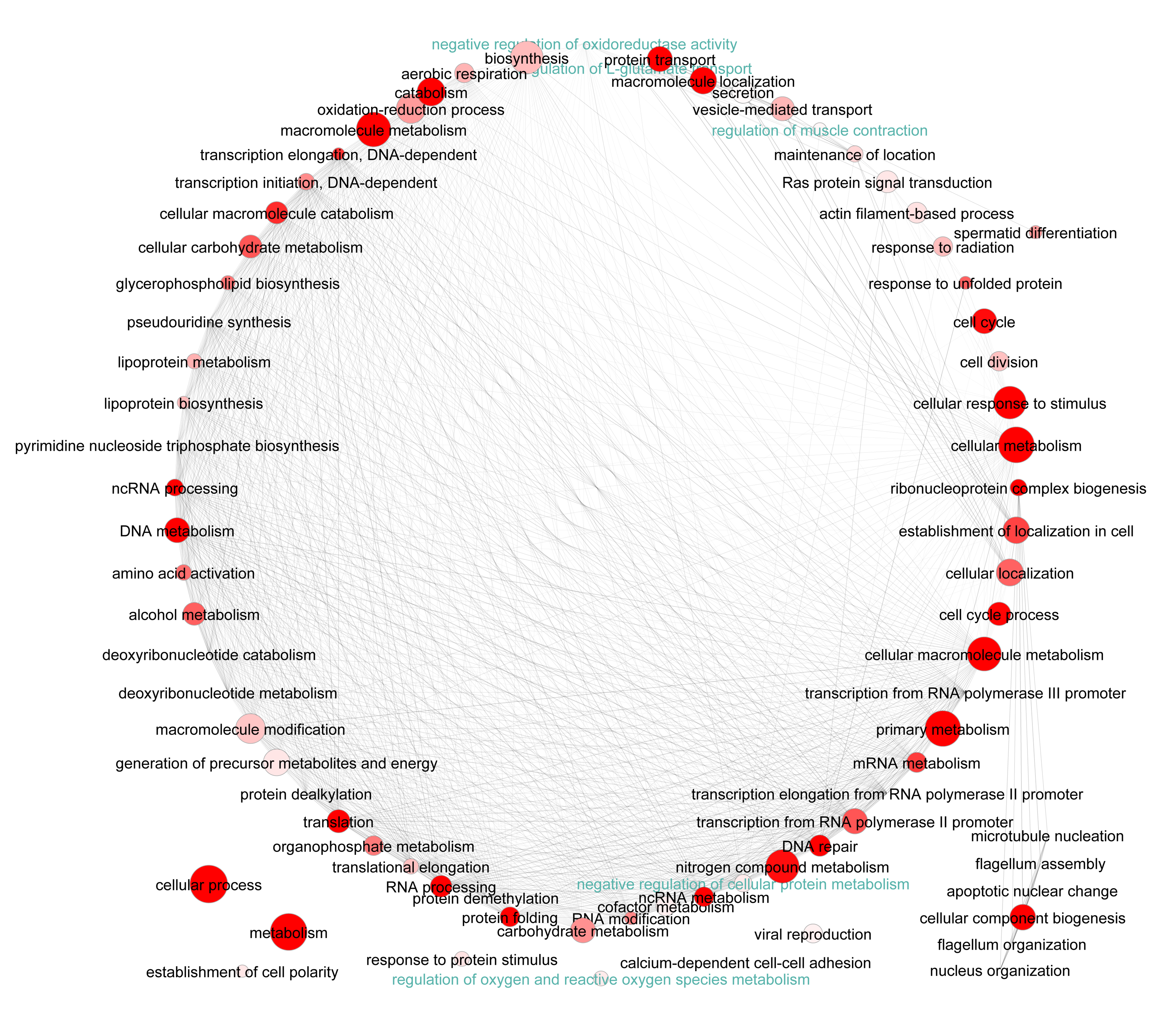

Supplement: Figure S8 — Over-represented GO terms for all protein-coding genes with neighbor genes within 5 kb in human. (TIF) [file pone.0052275.s008.tif]
