# Supplementary material for: Identification and Comparative Analysis of ncRNAs in Human, Mouse and Zebrafish Indicate a Conserved Role in Regulation of Genes Expressed in Brain
Source: PLoS One. 2012 Dec 20;7(12):e52275. doi: 10.1371/journal.pone.0052275 (PMC3527520; doi:10.1371/journal.pone.0052275)
Supplement: Table S2 — Summary of human and mouse known long ncRNAs that align to ESTs. This table contains a summary of human known long ncRNAs (chromatin-based, enhancer-like and RNA-seq based) and mouse long ncRNAs (chromatin-based, RNA-seq based) mapped against ESTs. (DOCX) [file pone.0052275.s010.docx]

**Table S2. Summary of human and mouse known long ncRNAs mapped to ESTs.**

| Name of long ncRNA dataset | Number of long ncRNAs | Number of ncRNAs mapped to raw ESTs | Number of ncRNAs mapped to quality cleaned, non-repeat ESTs |
| --- | --- | --- | --- |
| Chromatin based lincRNAs (human) | 4,860 | 2,224 (46%) | 1,590 (33%) |
| Enhancer like ncRNAs (human) | 3,011 | 2,981 (99%) | 2,654 (88%) |
| RNA-seq based ncRNAs (human) | 9,869 | 8,910 (90%) | 6,414 (65%) |
| Chromatin based lincRNAs (mouse) | 2,127 | 1,359 (64%) | 1,009 (47%) |
| *ES ncRNAs (mouse) | 591 | 572 (97%) | 440 (75%) |
| *NPC ncRNAs (moue) | 528 | 498 (94%) | 412 (78%) |
| *MLF ncRNAs (mouse) | 318 | 310 (97%) | 270 (85%) |

* These mouse long ncRNAs datasets are based on RNA-seq reconstruction form 3 different mouse cell types.
