# Supplementary material for: Identification and Comparative Analysis of ncRNAs in Human, Mouse and Zebrafish Indicate a Conserved Role in Regulation of Genes Expressed in Brain
Source: PLoS One. 2012 Dec 20;7(12):e52275. doi: 10.1371/journal.pone.0052275 (PMC3527520; doi:10.1371/journal.pone.0052275)
Supplement: Material S1 — Supporting results. (DOCX) [file pone.0052275.s012.docx]

**Evidence for transcription of previously annotated long ncRNAs based on ESTs**

We observed that there were only a small number of overlaps between our EST-based ncRNAs with previously annotated long ncRNAs. We used EST datasets from two different steps of our pipeline to check for evidence of transcription of these previously annotated long ncRNAs (Table 6)[1,2,3,4,5]. The first dataset contained the raw EST data that we used as a starting point for the identification of ncRNAs; 8,313,646 human ESTs and 4,850,846 mouse ESTs. The second dataset consisted of quality filtered and repeat masked ESTs; 5,675,245 human ESTs and 3,653,642 mouse ESTs. The numbers of ESTs in these datasets are smaller due to quality filtration and removal of ESTs with >50% repeat coverage

The alignments of previously known long ncRNAs against the EST datasets were carried out using BLASTN (e-value < 1e-3). A summary of these local alignment results is shown in Table S2. We observed that most enhancer like and RNA-seq based ncRNAs aligned well to ESTs, whether or not they contained repeats ESTs. However, the exons of chromatin-based lincRNAs, previously validated by tiling arrays, were less well aligned to ESTs. We analysed the tiling arrays (GPL8791 and GPL8792) that were used to detect lincRNA exons from K4-K36 domains and checked the probes for repetitive elements. We found that more than one third of the probes (125,758 out of 326,005 in array GPL8791 and 128,892 out of 348,261 in array GPL8792 based on hg19) originated from human repeats in both tiling arrays.

References:

1. Khalil AM, Guttman M, Huarte M, Garber M, Raj A, et al. (2009) Many human large intergenic noncoding RNAs associate with chromatin-modifying complexes and affect gene expression. Proc Natl Acad Sci U S A 106: 11667-11672.

2. Orom UA, Derrien T, Beringer M, Gumireddy K, Gardini A, et al. (2010) Long noncoding RNAs with enhancer-like function in human cells. Cell 143: 46-58.

3. Cabili MN, Trapnell C, Goff L, Koziol M, Tazon-Vega B, et al. (2011) Integrative annotation of human large intergenic noncoding RNAs reveals global properties and specific subclasses. Genes Dev 25: 1915-1927.

4. Guttman M, Amit I, Garber M, French C, Lin MF, et al. (2009) Chromatin signature reveals over a thousand highly conserved large non-coding RNAs in mammals. Nature 458: 223-227.

5. Guttman M, Garber M, Levin JZ, Donaghey J, Robinson J, et al. (2010) Ab initio reconstruction of cell type-specific transcriptomes in mouse reveals the conserved multi-exonic structure of lincRNAs. Nat Biotechnol 28: 503-510.
